# Raising the suspicion of a non-autochthonous infection: identification of *Leishmania guyanensis* from Costa Rica exhibits a Leishmaniavirus related to Brazilian north-east and French Guiana viral genotypes

**DOI:** 10.1590/0074-02760220162

**Published:** 2023-01-16

**Authors:** Carlos Mata-Somarribas, José Quesada-López, María F Matamoros, César Cervantes-Gómez, Annia Mejía, Karen Chacón, Ivannia Bendig, Roger Campos, Raphael Quesada-Morera, Lilian Motta Cantanhêde, Luiza de Oliveira R Pereira, Elisa Cupolillo

**Affiliations:** 1 Instituto Costarricense de Investigación y Enseñanza en Nutrición y Salud, Centro Nacional de Referencia de Parasitología, Cartago, Costa Rica; 2 Caja Costarricense de Seguro Social, Área de Salud Santa Rosa de Pocosol, Alajuela, Costa Rica; 3 Caja Costarricense de Seguro Social, Hospital Escalante Pradilla, San José, Costa Rica; 4 Caja Costarricense de Seguro Social, Hospital William Allen, Cartago, Costa Rica; 5 Caja Costarricense de Seguro Social, Área de Salud Florencia, Alajuela, Costa Rica; 6 Caja Costarricense de Seguro Social, Hospital Ciudad Neily, Puntarenas, Costa Rica; 7 Caja Costarricense de Seguro Social, Hospital de Guápiles, Limón, Costa Rica; 8 Caja Costarricense de Seguro Social, Área de Salud Matina, Limón, Costa Rica; 9 Caja Costarricense de Seguro Social, Área de Salud Talamanca, Limón, Costa Rica; 10 Fiocruz-Fundação Oswaldo Cruz, Instituto Oswaldo Cruz, Laboratório de Pesquisa em Leishmanioses, Rio de Janeiro, RJ, Brasil

**Keywords:** Leishmaniavirus, leishmaniasis, Central America, genetic relationship

## Abstract

**BACKGROUND:**

Costa Rica has a history of neglecting prevention, control and research of leishmaniasis, including limited understanding on *Leishmania* species causing human disease across the country and a complete lack of knowledge on the *Leishmania* RNA virus, described as a factor linked to the worsening and metastasis of leishmanial lesions.

**OBJECTIVES:**

The aim of this work was to describe a case of cutaneous leishmaniasis by *Leishmania* (*Viannia*) *guyanensis,* bearing infection with Leishmaniavirus 1 (LRV1) in Costa Rica, raising the suspicion of imported parasites in the region.

**METHODS:**

The *Leishmania* strain was previously identified by routine *hsp70* polymerase chain reaction-restriction fragment length polymorphism (PCR-RFLP) in Costa Rica and subsequently characterised by isoenzyme electrophoresis and Sanger sequencing in Brazil. Screening for LRV1 was conducted with a dual RT-PCR approach and sequencing of the fragment obtained.

**FINDINGS:**

Since 2016 Costa Rica performs *Leishmania* isolation and typing as part of its epidemiological surveillance activities. Amongst 113 strains typed until 2019, only one was characterised as a *L.* (*V.*) *guyanensis*, corresponding to the first confirmed report of this species in the country. Interestingly, the same strain tested positive for LRV1. Sequencing of the viral *orf1* and *2*, clustered this sample with other LRV1 genotypes of South American origin, from the Northeast of Brazil and French Guiana.

**MAIN CONCLUSION:**

The unique characteristics of this finding raised the suspicion that it was not an autochthonous strain. Notwithstanding its presumed origin, this report points to the occurrence of said endosymbiont in Central American *Leishmania* strains. The possibility of its local dispersion represents one more challenge faced by regional health authorities in preventing and controlling leishmaniasis.

Leishmaniasis is a complex of vector-borne diseases caused by flagellated protozoa of the genus *Leishmania* (Trypanosomatida: Trypanosomatidae). Globally, leishmaniasis is among the top 10 neglected tropical diseases, and continues to be a major health problem in four eco-epidemiological regions of the world: the Americas, East Africa, North Africa and West and Southeast Asia. In 2020, of the 200 countries and territories that reported to World Health Organization (WHO), 98 were considered endemic and six of having previously reported cases of leishmaniasis. In 2020, 208,357 new cutaneous leishmaniasis (CL) cases and 12,838 new visceral leishmaniasis (VL) cases were reported, with a mean number of deaths of approximately 500 cases.^1^ Infection with *Leishmania* spp, mainly recognised as a zoonotic disease, can cause a set of clinical syndromes in humans that compromise the skin, mucous membranes or viscera. Its complex transmission cycle includes different species of parasites, reservoirs and vectors. In the Americas specifically, they appear with high magnitude and wide distribution.^2^

The genus *Leishmania* shows biological complexities that are still not fully understood. Despite efforts to unravel the mechanisms of *Leishmania* pathogenicity, there’s an abundance of information from a multiplicity of different factors, the actual relevance of which is not completely determined. Some authors report that different *Leishmania* species and vectors, genetic traits and the immune condition and response of the host represent elements actively involved in the worsening of clinical presentations.^3^ ^-^ ^6^ ^)^ *Leishmania* parasites have been known to harbour an endosymbiotic *Leishmania* virus since the 1980s, and this relationship has been more recently studied, looking to explain the effects of this virus on *Leishmania* cells and its clinical and epidemiological consequences.^7^ ^-^ ^11^

Since the first reports on the existence of a *Leishmania* RNA virus 1 (LRV1), it has been speculated that its presence might affect the host-parasite interaction, causing changes in the virulence and pathology of the disease. The finding of LRV1 in *Leishmania* species associated with mucocutaneous forms of the disease, raised the suspicion that this endosymbiont could influence some aspects of its pathology.^12^ ^,^ ^13^

In Costa Rica, leishmaniasis has been considered one of the 10 most frequent notifiable diseases,^14^ with persistent hotspots of elevated risk throughout the country and an average incidence of 20,3 per 100,000 inhabitants from 2006 to 2017.^15^ The Regional Information System on Leishmaniasis in the Americas (SisLeish) applies the Composite Index of Leishmaniasis to assess the progress of each country’ disease-control program, according to the guideline of the “Action Plan on Leishmaniasis in the Americas 2017-2022”, which grouped Costa Rica with other high index countries for the 2016-2018 triennial.^16^

Nevertheless, in this Central American country, leishmaniasis represents a clear example of a neglected disease. Its high incidence and particular epidemiology reveal a prevalent infection with high transmission rates in the local population, with the common appearance of severe clinical presentations, treatment failure and drug resistance.^14^ ^,^ ^17^ ^,^ ^18^ ^,^ ^19^ ^)^ Costa Rica’s limitations in managing and preventing this disease reveal a historical vacuum of relevant epidemiological information and a void in technical and scientific knowledge of the infection. The information available to the public health authorities and scientific community, for decision-making, is very limited.

Since 2016, the Instituto Costarricense de Investigación y Ensenanza en Nutrición y Salud (Inciensa)*,* as the national parasitological reference laboratory, types all *Leishmania* parasites isolated from patients presenting CL by *hsp70* polymerase chain reaction-restriction fragment length polymorphism (PCR-RFLP).^20^ ^,^ ^21^ Of note was the detection of *Leishmania* (*Viannia*) *guyanensis* in one patient since this species has not been observed in the country so far. The association of this species with LRV1 led us to investigate the presence of this viral endosymbiont in this strain. Here we will present some details about the first report of *L.* (*V.*) *guyanensis* and LRV1 in Costa Rica, discussing possible implications of this finding.

## MATERIALS AND METHODS

*Study design and Leishmania strains -* The Centro de Referencia de Parasitología (CNRP) of the Inciensa*,* in Cartago, Costa Rica, as the national parasitological reference laboratory, collected and isolated *Leishmania* spp strains over the years, as part of its strategy of epidemiologic surveillance. Every sample collection was conducted after appropriate patient briefing and signed consent form. Procedures are in agreement with the Helsinki Declaration of 1975 and revised in 1993. In 2016, a protocol for *Leishmania* species typing based on PCR-RFLP^20^ ^,^ ^21^ ^)^ was implemented at CNRP. Until 2019, 113 samples had been collected and typed, but for technical reasons only 24 strains were available after cryopreservation and were transferred to the Coleção de Leishmania, Instituto Oswaldo Cruz, Fundação Oswaldo Cruz (CLIOC)*,* in accordance with its regulations. Table sum­marises the information from the 24 strains. 

All samples were kept in culture for the period of the analysis with weekly or biweekly passages, in accordance with the CLIOC standardised procedures and protocols.^22^

**TABLE:** Clinical and demographic information on the strains of *Leishmania* spp evaluated in this study

Sample	Patient		Geographic location in Costa Rica			Clinical presentation	Species	Date of isolation
	Date of birth	Gender	District	Canton	Province			
1	31 Dec 2000	Female	La Tigra	San Carlos	Alajuela	LC	*L. (Viannia)* sp*.*	19 Jul 2018
2	29 Aug 1994	Male	Corredor	Corredores	Puntarenas	LC	*L. (Viannia)* sp*.*	24 Jul 2018
3	27 Sep 1963	Female	Guápiles	Pococi	Limón	LC	*L. panamensis*	31 Jul 2018
4	28 Sep 1997	Female	Batán	Matina	Limón	LC	*L. panamensis*	24 Aug 2018
5	27 Oct 1980	Male	Matina	Matina	Limón	LC	*L. panamensis*	06 Sep 2018
6	12 Dec 1987	Female	Batán	Matina	Limón	LC	*L. panamensis*	17 Oct 2018
7	27 Jul 1987	Male	Carrandí	Matina	Limón	LC	*L. panamensis*	02 Oct 2018
8	20 Jan 1999	Male	Barú	Pérez Zeledón	San José	LC	*L. (Viannia)* sp*.*	01 Nov 2018
9	05 Feb 1999	Female	Pejibaye	Pérez Zeledón	San José	LC	*L. (Viannia)* sp*.*	06 Nov 2018
10	18 Jan 2008	Female	Barú	Pérez Zeledón	San José	LC	*L. panamensis*	12 Nov 2018
11	05 Feb 1954	Male	Pejibaye	Pérez Zeledón	San José	LC	*L. panamensis*	26 Nov 2018
12	25 Feb 1956	Male	Río Nuevo	Pérez Zeledón	San José	LC	*L. (Viannia)* sp.	26 Nov 2018
13	08 May 1957	Male	Pejibaye	Pérez Zeledón	San José	LC	*L. panamensis*	19 Dec 2019
14	17 May 1999	Male	Pejibaye	Pérez Zeledón	San José	LC	*L. panamensis*	13 Dec 2019
15	28 Dec 1959	Male	Pejibaye	Pérez Zeledón	San José	LC	*L. panamensis*	27 Dec 2019
16	29 Jan 1953	Male	Turrialba	Turrialba	Cartago	LC	*L. panamensis*	31 Oct 2018
17	Unknown	Female	Jimenez	Pococi	Limón	LC	*L. panamensis*	10 Jan 2019
18	16 Jul 1988	Male	Corredor	Corredores	Puntarenas	LC	*L. panamensis*	11 Feb 2019
19	19 Dec 1992	Male	Cutris	San Carlos	Alajuela	LC	*L. guyanensis*	12 Feb 2019
20	26 Mar 1983	Male	Telire	Talamanca	Limón	LC	*L. (Viannia)* sp.	26 Apr 2019
21	09 Aug 1985	Female	Cahuita	Talamanca	Limón	LC	*L. panamensis*	25 Apr 2019
22	01 Oct 1995	Male	Batán	Matina	Limón	LC	*L. panamensis*	16 Apr 2019
23	31 Oct 2007	Male	Batán	Matina	Limón	LMC	*L. panamensis*	21 Mar 2019
24	14 Jun 1982	Male	Lepanto	Puntarenas	Puntarenas	LC	*L. braziliensis*	28 Mar 2019

CL: cutaneous leishmaniasis; MCL: mucocutaneous leishmaniasis.

*Isoenzyme electrophoresis -* Following the routine of CLIOC, the 24 strains were typed by isoenzyme electrophoresis and *hsp70* sequencing before cryopreservation and storage. The isoenzyme electrophoresis methodology is already used in the CLIOC routine, with standardised and internationally accepted protocols^23^ ^,^ ^24^ performed for two enzyme systems capable of distinguishing the main species circulating in the Americas: glucose-6-phosphate dehydrogenase (G6PDH, E.C.1.1. 1.49) and 6-phosphogluconate dehydrogenase (6PGDH, E.C.1.1.1.44).

*Hsp70 sequencing protocol -* For *hsp70* amplification by PCR and Sanger-sequencing, specific primers and protocol were used based on standardised and internationally accepted procedures.^25^ The amplified products were purified using the MinElute 96 UF PCR Purification Kit (28053 Qiagen, Hilden, Germany), following the manufacturer’s instructions. The samples were delivered to the Genomics Platform - DNA sequencing/PDTIS-Fiocruz for processing in an ABI 3730 DNA Analyzer automatic sequencer (Applied Biosystems, Foster City, USA). The MEGA X (Molecular Evolutionary Genetics Analysis version 10.2.2, Pennsylvania State University, State College, USA), and BioEdit (BioEdit Sequence Alignment Editor version 7.2.5, Tom Hall) software were used to edit and align the sequences, respectively. The groupings of species, or “clustering”, were obtained both through the construction of networks using the NeighborNet method in the Splitstree program (SplitsTree4 version 4.17.1, Eberhard Karls University of Tübingen, Tübingen, Germany) and with the Barcode gap species delimitation program Automatic Barcode Gap Discovery (ABGD web version 08/26/21).

*RNA extraction, cDNA synthesis -* RNA was isolated from at least 1 × 10^7^ promastigotes of every strain in the exponential growth phase, with 1 mL of Trizol TRI Reagent^®^ (T9424, Sigma-Aldrich, St. Louis, USA) according to the manufacturer’s instructions and protocol. RNA was quantified using a Nanodrop 1000 spectrophotometer (Thermo Scientific, Wilmington, USA). A total of 2 μg of RNA was treated with DNAase with the RQ1 RNase-Free DNase protocol (M6101 Promega, Madison, USA). cDNA was obtained applying the High-Capacity cDNA Reverse Transcription Kit (4368814, Applied Biosystems) according to the manufacturer’s instructions and protocol. The cDNA was used in the detection of LRV by two different PCR systems.

*Dual RT-PCR approach -* A nested RT-PCR was initially used to test the samples, according to previously published protocols.^26^ A second RT-PCR with a longer product was used to confirm the findings of the first RT-PCR, also using protocols previously published.^27^ ^)^ A *L.* (*V.*) *guyanensis* LRV1 positive isolate (*L.* (*V.*) *guyanensis* - MHOM/BR/1975/M4147) was used as a positive control. Leishmanial actin was used as a control gene.^26^ ^)^ Aliquots of the PCR products were subjected to 2% agarose gel electrophoresis for visual confirmation of PCR amplification under ultraviolet light. Assays were repeated using experimentally distinct samples obtained from different RNA extractions to confirm the results. 

*LRV1 sequencing protocol -* The LRV1 sequencing protocol was as previously described for *hsp70*. To assess the relationship between sample and reference strains we produced distance matrices and trees where clusters were formed in agreement to each sequence similarity. The distance between sequences was determined using the p-distance test,^28^ in the MEGA program. Clustering was assessed through the construction of trees by the neighbour-joining method^29^ ^)^ using 1,000 bootstrap replicates,^30^ comprising the *orf* 1 and 2 sequences of the LRV genome regions that allows viral classification. 

## RESULTS

**Fig. 1:**
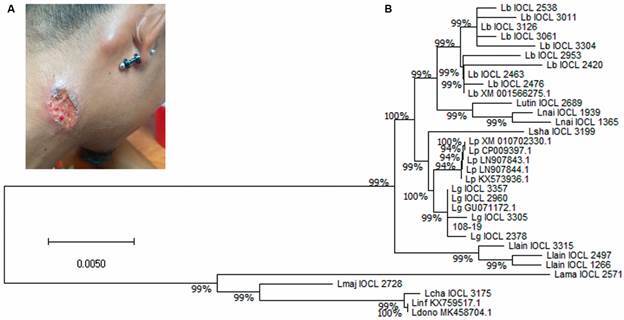
lesion appearance and parasitic classification of the isolated and cultivated *Leishmania* strain. A) Image of cutaneous lesion and nearby area, noticing the lymph node involvement. B) Neighbour-joining tree based on genetic distances (p-distance) between each pair of sequence for the leishmanial *hsp70* region. Reference strains were obtained from publicly available leishmanial sequences and compared to the positive sample, marked with a black arrow (GenBank^®^ accession ON075820).

Of the 24 *Leishmania* strains typed at Inciensa and CLIOC, only one (internal code #108-19/CLIOC code IOCL3804) was positive after LRV1 examination, the same one typed as *L.* (*V.*) *guyanensis*, a not yet identified etiological agent of CL in Costa Rica. This strain was isolated from a male patient, 29 years of age, from one of the northernmost regions of the country, Cutris de San Carlos, of the Alajuela province. On the right side of the neck the patient had a 5 cm (Fig. 1A), 3 months old, deep ulcer, with pruritic and sanguine-purulent tissue. Below his right ear, and above this ulcer, he had a visibly swollen lymph node. No other symptom was reported. The diagnosis was confirmed locally at the Area de Salud de Santa Rosa de Pocosol, through light microscopy of a direct smear, and the stained slide, along with culture media seeded with lesion aspirate, were send to Inciensa for confirmation and species characterisation. This strain was identified as *L.* (*V.*) *guyanensis* by isoenzyme electrophoresis, routinely used for *Leishmania* typing by the CLIOC^23^ and confirmed by sequencing of a *hsp70* fragment amplified by PCR.^25^ The obtained sequence (GenBank^®^ accession ON075820) was aligned with 32 other *Leishmania hsp70* sequences retrieved from GenBank and clearly clustered with *L.* (*V.*) *guyanensis* sequences (Fig. 1B).

**Fig. 2:**
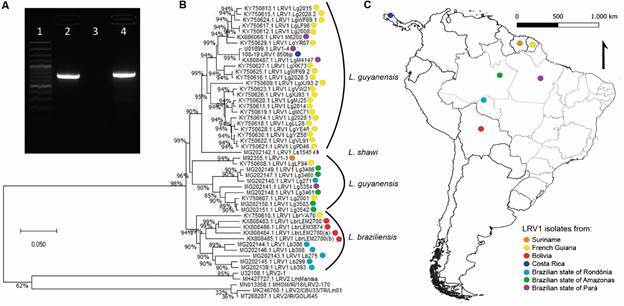
RT-PCR products, genetic distance and colour-coded geographic grouping between *Leishmania* RNA virus (LRV) sequences. A) RT-PCR fragment of the *orf* 1 and 2 regions (850 bp) lane 1: size marker, 100-1,000 base pairs (bp); 2: *Leishmania* (*Viannia*) *guyanensis* M4147; 3: negative control; 4: sample #108-19. In the size ladder, the more intense bands correspond to 1,000 bp and 500 bp, from top to bottom. B) Neighbour-joining tree based on genetic distances (p-distance) between each pair of sequences, for the *orf* 1 and 2 fragments of LRV. Corresponding *Leishmania* species can be seen to the right of the tree. Reference strains were obtained from publicly available LRV1 and two sequences and compared to the positive sample (GenBank^®^ accession OM140825). Colour-coded dots correspond to Fig. 1C colour code. C) Grouping of samples by geographic location, either country of origin or state of origin in the case of Brazilian samples. The colour code can be seen in the bottom right corner.

One typical RT-PCR product gel is shown in Fig. 2, illustrating the confirmation of one, out of the 24 strains, as positive for the endosymbiont. Nested LRV RT-PCR and parasitic actin tests are not show herein. The amplified product chosen for sequencing was the longest one (corresponding to Fig. 2A), since this fragment displays approximately 850 base pairs (bp) corresponding to part of the *orf1* region and the beginning of the *orf2* region, including the portion responsible for encoding the viral capsid protein.^27^

Assessing the relationship between sample and reference strains we produced a tree (Fig. 2B) that shows two distinct groups separating the main *Leishmania* (*Viannia*) species harbouring virus: *L.* (*V.*) *guyanensis* and *L.* (*V.*) *braziliensis.* The #108-19 sample clustered with the LRV1 sequences from *L.* (*V.*) *guyanensis* strains, confirming the identification of this strain, which was previously characterise through isoenzyme electrophoresis and *hsp70* sequence analysis. The #108-19 LRV1 sequence grouped with other LRV1 sequences from the Northeast of the Brazilian Amazon region and from the French Guiana (Fig. 2C). It was more distantly related to sequences from the Amazonas and Rondônia states of Brazil (northwest Amazonian region) and Bolivia, which could indicate a geographic association. 

## DISCUSSION

There’s a high probability that the *L.* (*V.*) *guyanensis* strain isolated from a Costa Rican patient could represent an imported case from a neighbouring country and not an autochthonous *Leishmania* strain. This particular patient claimed to be an informal construction worker. Additional information on the lesion’s evolution and treatment response was unobtainable since the patient couldn’t be located afterwards by public health authorities, giving rise to suspicions about the means of his livelihood. During this time, there was a significant illegal mining activity in nearby areas, particularly Crucitas near the Nicaraguan border*,* conducted primarily by Nicaraguan illegal immigrants, with reported cases of CL. The northern border of Costa Rica represents a porous frontier through which thousands of temporal workers and migrants, especially those making their way to the United States, pass through, including people of Asian, African and Latin American descent. Costa Rica is still seen as a “humanitarian transit country”, which does not formally engage in border securitisation, at least not at the same level as its regional neighbours. People who flee from violence and poverty in countries such as Venezuela, Colombia and Nicaragua generate new groups of refugees.^31^ Costa Rica has also been an important Central American hub of recently increased African and Asian transit migration across Latin America, directly from their home countries or from in-between destinations such as Brazil.^32^ ^)^ Migrants are known for their continuous movement through the border, taking part in temporary jobs on this side of the crossing. Moreover, there has never been a report, until now, of a *L.* (*V.*) *guyanensis* strain as the one characterised in this instance, whose clustering and sequence similarities indicate a classic genotype of *L.* (*V.*) *guyanensis* (Fig. 1B), not previously seen in the country*.* War, armed conflict, natural catastrophes or any other agent of human migration can generate the introduction of non-autochthonous or rare species of *Leishmania*, giving the ability of communicable diseases as leishmaniasis to cross borders.^33^ Indigenous sandflies, if vectorially competent, could introduce “new” *Leishmania* species from infected humans into domestic and wild animals, facilitating the establishment of reservoirs and complicating control efforts.^34^

The strain identified as *L.* (*V.*) *guyanensis* was also positive for LRV1. The relationship between *Leishmania* and this endosymbiont began to unravel when virus-like particles with no lytic replication nor clear infectivity were described in different species of *Leishmania.* ^35^ ^,^ ^36^ The virus was named LRV, from *Leishmania* RNA virus.^12^ The lack of a detectable infectious phase suggests a long-lasting relationship between the virus and *Leishmania*, this was corroborated by similar genetic intervals between the parasite and LRV, signifying a symbiotic association^37^ ^)^ established prior to its divergence between Old and New World strains, sustaining the hypothesis that LRV is an ancient virus of *Leishmania* spp and probably spread following host diversification,^38^ ^)^ which justifies its findings throughout the region and its appearance in Central America.

The finding of only one positive strain represents a much lower percentage, comparable only with that of Pereira et al.,^26^ than the average study regarding LRV, which shows a positivity that could be established at around 37%.^11^ Nevertheless, it is important to remember that this report constitutes the first one comprising solely Central American strains, with an unknown prevalence of the virus.

It has been described that the *Leishmania* culture process has the limitation of producing artificial biases.^39^ ^,^ ^40^ Thus, the selection of clones with a better aptitude for growth under culture conditions may change the original variability of the strain, same as a sampling bias may initially have selected only particular individuals from the original population. *Leishmania* strains have been shown to cocultivate LRV positive and negative parasites with different survival times and responses to environmental stress during *in vitro* cultivation. Different parasite mechanisms are suspected of this particular interaction such as cell-cell contact and secretion factors, such as exosome secretion, recently demonstrated for LRV1+ parasites,^38^ ^,^ ^41^ giving *Leishmania* the ability to synthesise and secrete compounds in the shared environment, affecting population density and parasite behaviour. Given the fact that no RNA extraction was performed directly from the lesion site, that the strains were sent to CLIOC in media culture already isolated and that these were kept through numerous passages, the Costa Rican samples could had suffered from an artificial selection. 

LRV1 has been previously reported in a *Leishmania* sp. strain from Costa Rica, albeit indirectly.^42^ In that study, the authors compared LRV1- and LRV1+ strains in terms of immune response, the latter producing a predominant Th2-biased response, which was correlated in humans to poorer immune control of infection and more severe disease. Their report included two *L.* (*V.*) *panamensis* from Costa Rica producing severe disease. One of these was LRV+, comprising the first report of this endosymbiont on a Costa Rican *Leishmania* strain. *Leishmania* spp infection carries an important risk for metastasis and the development of complicated and difficult-to-treat secondary lesions, with mucocutaneous leishmaniasis being a common outcome.^43^ In that context, the presence of LRV has been determined as a potent innate immunogen, redirecting the immune response of the host by inducing a hyper-inflammatory reaction and possibly triggering dissemination,^9^ allowing the repeated metastasis of LRV+ parasites, in contrast with LRV1- parasites. It has been associated also with mucosal leishmaniasis in humans,^10^ ^)^ with an increasing risk of therapeutic failure^44^ ^)^ and with first-line treatment failure and relapse.^45^ This is particularly relevant in this report as the clinical presentation of the case matches the descriptions associated with an LRV+ strain: the lymph node involvement seen here is associated with more severe presentations of the disease. 

It is expected that *Leishmania* harbouring LRV could display better performance and fitness than virus-free strains facing certain environmental challenges.^38^ ^)^ It seems clear that a set of diverse factors must be involved in the worsening of a leishmanial lesion, but it is a fact that the presence of LRV1 affects the parasite pathogenic potential. The existence of this viral endosymbiont, due to its coevolution process with *Leishmania*, with the risk of worsening the clinical outcome, represents another challenge to clinicians and local, or regional, laboratories in *Leishmania-*endemic areas. Specialised laboratory techniques in national reference centres should be available for the appropriate identification, characterisation and follow-up of these cases. 

The data reported here points to the importance of epidemiological surveillance strategies in the region. The clinical case described herein, associated with the species *L.* (*V.*) *guyanensis*, could be imported, nevertheless little is actually known about the possibility of the establishment of this species in the region. The fact that the LRV1 endosymbiont has also been found, adds to this concern. Literature on this topic already shows therapeutic failure when antimonials are the drug of choice for *L.* (*V.*) *guyanensis* infections,^46^ ^,^ ^47^ and recently, the use of miltefosine has been strongly recommended by Pan American Health Organization (PAHO) to treat adult patients diagnosed with CL caused by *L*. (*V.*) *guyanensis*, for example.^48^ However, *Leishmania* species and the presence of LRV1 are still not yet considered for administration of treatment in many countries, which is the case for Costa Rica.
